# Integrated analysis of recurrent properties of cancer genes to identify novel drivers

**DOI:** 10.1186/gb-2013-14-5-r52

**Published:** 2013-05-29

**Authors:** Matteo D'Antonio, Francesca D Ciccarelli

**Affiliations:** 1 Department of Experimental Oncology, European Institute of Oncology, IFOM-IEO Campus, Via Adamello 16, 20139 Milan, Italy

**Keywords:** Driver mutations, cancer genetic heterogeneity, interaction network, gene duplication, gene origin, gene expression

## Abstract

The heterogeneity of cancer genomes in terms of acquired mutations complicates the identification of genes whose modification may exert a driver role in tumorigenesis. In this study, we present a novel method that integrates expression profiles, mutation effects, and systemic properties of mutated genes to identify novel cancer drivers. We applied our method to ovarian cancer samples and were able to identify putative drivers in the majority of carcinomas without mutations in known cancer genes, thus suggesting that it can be used as a complementary approach to find rare driver mutations that cannot be detected using frequency-based approaches.

## Background

In recent years, the completion of dozens of high-throughput sequencing screenings of cancer genomes led to the identification of >10,000 genes that bear at least one non-synonymous mutation. The discovery of such a wealth of mutations that progressively accumulate in the cancer genome was to some extent surprising and substantiated the idea of tumours as evolutionary systems where most acquired variations are 'passenger' because they do not have any direct role in promoting cancer. These mutations are fixed in the cancer cell population owing to the presence in the same cells of 'driver' mutations that instead confer growth advantages [1]. The identification of the (few) driver mutations among the (many) passenger variants is therefore key to pinpoint genes and pathways that play an active role in cancer development and may be used as therapeutic targets. Unfortunately, the distinction between driver and passenger mutations is not straightforward, because of the high heterogeneity of the mutational landscape among and within cancer types [2]. One of the most widely used approaches to identify novel cancer genes (that is, genes that harbour driver mutations) measures the gene mutation frequency, relying on the assumption that genes that are important for the development of a certain cancer type are recurrently mutated in several tumours [2-17]. Frequency-based methods led to the detection of unexpectedly high mutation frequency of isocitrate dehydrogenases 1 and 2, eventually linking these enzymes to the onset of leukaemia and glioma [12,18]. They also contributed to better understand the genetic heterogeneity of cancer, leading to the observation that only few genes are mutated in the vast majority of tumour types, while most cancer genes are mutated at high frequency in one or few cancer types [19]. Also the analysis of pathways instead of genes contributed to reduce the heterogeneity of cancer mutational landscape, because often the de-regulation of cancer-associated pathways can occur through the mutations of different components [20]. Pathway analysis for example identified significant enrichment of mutations in *BRCA1 *and *ATM *pathways in breast cancer, and *WNT *and *TGFβ *signalling pathways in colorectal cancer [21]. Although these processes were already known to be involved in tumorigenesis [20], only a systematic approach led to assign a likely driver role to new pathway components. Following conceptually similar approaches, several groups have analysed the proteins encoded by cancer genes in the context of the human protein-protein interaction network and identified network modules that are significantly associated with mutations [22-24]. Network analysis showed that cancer genes encode proteins that are highly connected and central inside the network [25,26]. This has been interpreted as a sign of fragility of cancer genes towards perturbations, because modifications of proteins at the crossroad of multiple biological processes are likely to have harmful consequences [27]. In addition to encoding highly connected and central proteins, cancer genes share also other systems-level properties (that is, global properties that do not strictly depend on the individual gene function) that distinguish them from the rest of human genes. For example, they tend to maintain only one single copy in the genome, which suggests an intrinsic sensitivity of cancer genes towards gene dosage imbalance [26]. Moreover, cancer genes mostly appeared at two time points in evolution: caretakers and tumour suppressors are ancient genes that have orthologs also in prokaryotes, while gatekeepers and oncogenes were acquired with metazoans [28]. This suggests that tumorigenesis may arise from the impairment of either very basic or regulatory processes [29]. The existence of properties that distinguish cancer genes from the rest of human genes may be used to discriminate between driver and passenger mutations because mutated genes that have properties similar to known cancer genes are, in principle, more likely to harbour driver mutations, particularly when the mutations alter the protein function. In the last years, several methods to predict damaging mutations have been developed taking into account the site conservation throughout evolution and the possible effects on protein structure, as well as on splice-sites and UTRs [30-34]. In this study we developed an integrative method that uses tumour, gene and mutation properties to eventually predict novel drivers. As a proof of principle we applied our selection procedure to a panel of >300 ovarian carcinoma patients and identified genes with a putative driver role in >70% of tumours with previously unknown genetic determinants.

## Results

### The mutational landscape is cancer-specific and recurrently mutated genes are long

**Figure 1:**
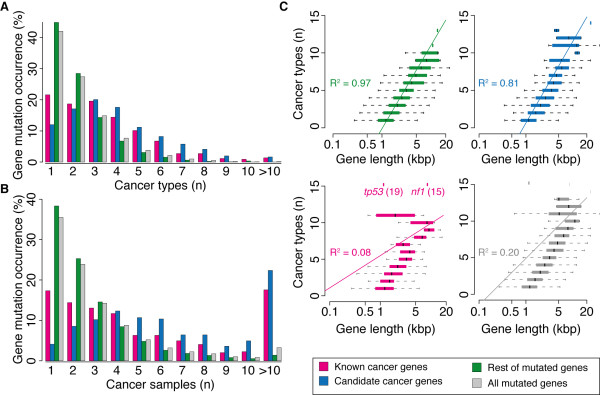
**Mutation occurrence and correlation with gene length of known, candidate and rest of mutated genes**. Occurrence of mutated genes in 20 cancer types (**A**) and 3,052 samples (**B**). None of the 10,681 genes is mutated in all 20 cancer types or samples; *TP53 *is the only gene to be mutated in 19 cancer types, while >40% of genes are mutated only in one cancer type. (**C**) Dependence of the recurrence of mutations on the gene length. Plotted is the length distribution of the coding portion for all genes that were found mutated in one to 20 cancer types. The interpolation line and R^2 ^were calculated using the LM function in R.

We collected 10,681 human genes with at least one non-synonymous mutation from 39 high-throughput mutational screenings conducted in 3,052 cancer samples and 20 cancer types [2,4-18,35-57]. We divided these mutated genes into three groups: (1) 444 known cancer genes that are part of the Cancer Gene Census, a literature-based collection of genes that play experimentally-proven driver roles in cancer development [58,59]; (2) 608 candidate cancer genes that are likely to play a driver role (see Additional file 2, Table S1 for the definition of candidates in each study); and (3) 9,629 genes with no evidence of active involvement in cancer (Table 1 and Additional file 2, Table S1). As already reported [2,27], we confirmed the heterogeneity of cancer mutational landscape and the overall tendency of genes to be mutated only in few cancer types (Figure 1A) and samples (Figure 1B). In particular, 40% of genes with no evidence of involvement in cancer are mutated in only one cancer type or sample, and <10% recur in more than four cancer types or samples (Figure 1A, B). This indicates the likely enrichment of these genes in passenger mutations. Similarly, the observed tendency of candidates to mutate in several samples (Figure 1B) likely reflects the frequency-based methods that were used to identify them (Additional file 2, Table S1).

**Table 1:** Dataset of known, candidates and mutated cancer genes

Cancer type	Known cancer genes	Candidate cancer genes	Rest of mutated genes	Tumour samples	References
Bladder	24	9	380	97	[8]

Breast	165	280	1,520	196	[2,11,40,52]

Colorectal	42	124	654	11	[2]

Gastric	203	14	5,160	109	[16]

Glioblastoma	127	87	1,679	197	[5,12,39]

HNSCC	221	67	5,901	194	[15,35]

Kidney	71	15	655	517	[7,9,55]

Leukaemia	58	169	877	393	[18,41,44,46,50,51]

Liver	26	66	370	140	[45,53]

Lung	218	143	1,012	324	[4,11,43,49]

Lymphoma	46	47	574	133	[14,47]

Medulloblastoma	16	1	194	88	[13]

Melanoma	96	159	2,845	16	[17,48,54]

Myelodysplasia	22	4	206	29	[57]

Myeloma	69	6	1,260	37	[6]

Oligodendroglioma	12	0	149	34	[38]

Ovarian	60	80	109	58	[11]

Pancreas	77	194	1,070	205	[10,11,42,56]

Prostate	54	161	81	67	[11,37]

Sarcoma	11	10	0	207	[36]

**Total**	**444**	**608**	**9,629**	**3,052**	NA

Starting from 10,681 human genes with at least one cancer-specific non-synonymous mutation, three groups were identified: known cancer genes that are part of the cancer gene census [58]; candidate genes that are likely to play a role in cancer because they are recurrently mutated; and the rest of mutated genes that carry at least one non-synonymous mutation. The full description of mutated genes in each of the 39 sequencing screenings is reported in Additional file 2, Table S1. HNSCC; head and neck squamous cell carcinoma.

Next, we checked whether gene length might influence the recurrent mutations of the same gene in multiple tumours, since longer genes are likely to host a higher number of mutations. Indeed we found positive correlation between the tendency of a gene to be recurrently mutated and its length, particularly in the case of mutated genes with no evidence of cancer involvement, but surprisingly also for candidates (Figure 1C). In both groups the vast majority of genes that are mutated in >10 cancer types have a coding portion longer than 4,450 bp (top 5% of the longest human genes). As a comparison, only five known cancer genes that are mutated in >10 cancer types (*NF1*, *EP300*, *BRCA2*, *MLL*, *ARID1A*) are longer than 4,450 bp. Although a positive correlation between gene length and the number of mutations was expected for genes harbouring passenger mutations, the fact that it was observed also for candidates, but not for known cancer genes, show that current methods do not completely correct for this effect.

Our survey of cancer somatic mutations confirmed that most of them are cancer- and sample-specific. Furthermore, gene length influences the recurrence of mutations and it should be taken into account when selecting candidates only on the basis of gene mutation frequency.

### The majority of mutated genes are tissue-selective and lowly expressed

**Figure 2:**
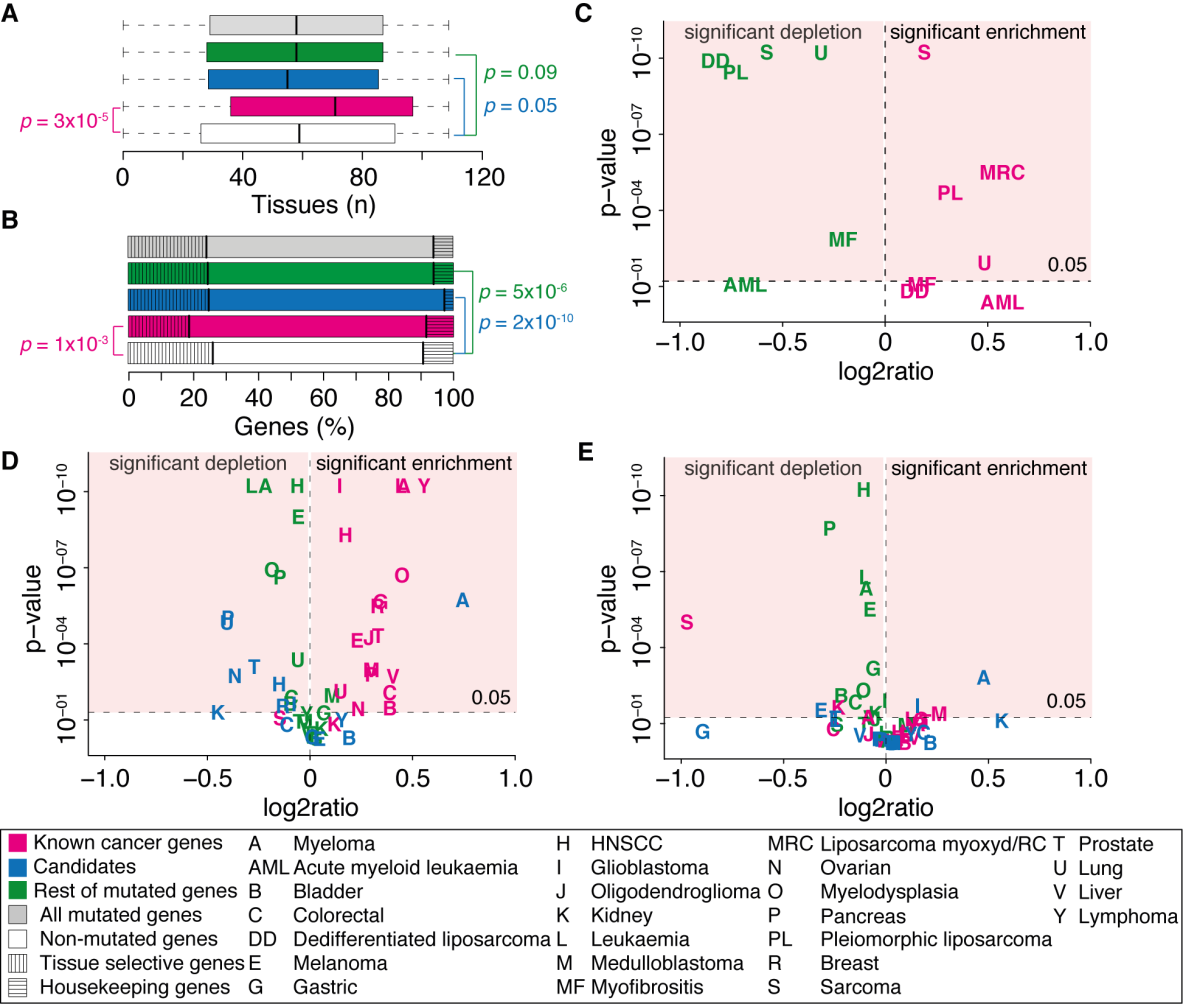
**Expression of known, candidate and rest of mutated genes in cancer and normal tissues**. (**A**) Breadth of expression of mutated genes in healthy tissues. Since the data were not normally distributed (*P *value 10^-42^, Shapiro-Wilk test, Additional file 1, Figure S1), distributions were compared using the Wilcoxon test. (**B**) Fraction of housekeeping and tissue-specific mutated genes. Housekeeping genes were defined as genes expressed in 107/109 tissues (98%). Tissue specific genes were defined as genes expressed in 27/109 tissues (<25%). Fisher's exact test with one degree of freedom was used to determine statistical significance. (**C**) Volcano plot showing the log2ratios between the fractions of expressed genes in each group of mutated genes and in non-mutated genes. For each log2ratio, the corresponding *P *value from the chi-squared test with one degree of freedom is also shown. None of the three studies used for this analysis [36,43,44] identified candidate cancer genes, thus only the expression of known cancer genes and other mutated genes could be checked. (**D**) Volcano plot showing the log2ratios between the fractions of mutated genes (known cancer genes, candidates and other mutated genes) and non-mutated genes that are expressed in the normal counterparts of the 20 tumour types. The *P *value from the chi-squared test, one degree of freedom for each log2ratio is also shown. For assignment of normal tissues to tumour types see Additional file 2, Table S3. (**E**) Volcano plot showing the log2ratios of the faction of highly expressed mutated genes compared with the rest of highly expressed human genes. Highly expressed genes were identified as those genes with expression higher than the median expression for that tissue (see Methods).

Indirect pieces of evidence have recently shown that gene expression may be useful for discriminating between driver and passenger mutations. For example, mutations of expressed genes in lung carcinomas are overall negatively selected, while the mutation rate of non-expressed genes is similar to the genome-wide average [43]. Based on this observation we reasoned that mutations affecting coding sequences are more likely to exert their function if the gene is expressed. To check whether this is true we investigated the breadth of expression (that is, the number of tissues where a gene is expressed) of mutated genes in a panel of 109 healthy human tissues. Overall we found that known cancer genes are expressed in a significantly higher number of tissues than non-mutated human genes, while candidates and other mutated genes show narrow expression breadth (Figure 2A, Additional file 1, Figure S1, Wilcoxon test). Moreover, known cancer genes are significantly depleted in tissue selective genes (that is, genes expressed in <25% of the total, Fisher's exact test), while candidates and other mutated genes are significantly depleted in housekeeping genes (that is, genes expressed in at least 98% of the total, Figure 2B, Fisher's exact test). These results confirm that known cancer genes are housekeeping and broadly expressed.

We further investigated whether mutated genes are expressed in the same tissues where they are mutated. Unfortunately, such a direct comparison was possible only for three studies that had both mutation and expression data on the same samples, including the whole genomes of acute myeloid leukaemia [44] and primary lung tumour [43], and the mutational screenings of 722 protein-coding genes in 207 sarcoma samples [36]. In all three studies we found a clear distinction between known cancer genes, which are expressed in higher fraction than the rest of human genes, and other mutated genes, which instead are expressed in lower fraction (Figure 2C, Additional file 2, Table S2, chi squared test). To confirm that this is a general trend in all 20 cancer types with available mutation data, we checked for the expression of mutated genes in the corresponding healthy counterparts (Additional file 2, Table S3). We found that in the normal tissues corresponding to 18 of the 20 cancer types, the fraction of expressed known cancer genes is higher than the rest of expressed human genes, and in 14 cases this difference is statistically significant (Figure 2D and Additional file 2, Table S4, chi squared test). The majority of both candidates and other mutated genes are instead not expressed in the tissues where they were found mutated (Figure 2D, Additional file 1, Figure S2A, chi-squared test). The only significant exception were candidate cancer genes in myeloma, which were expressed in higher fraction than the rest of human genes, probably also because of an overall low expression of human genes in blood and bone marrow (Additional file 2, Table S4). Interestingly, even when mutated genes are expressed, their expression levels are lower than the median expression of non-mutated genes in the same tissues, while known or candidate cancer genes are expressed at levels comparable with the overall tissue median (Figure 2E, Additional file 1, Figure S2B, and Additional file 2, Table S5, chi-squared test).

Altogether, these data showed that cancer genes with driver mutations tend to be expressed in the tissue where they are mutated, while genes likely harbouring passenger mutations are generally not expressed. Expression can be therefore used as a further filter to distinguish passenger from driver mutations. Although this might be expected, so far gene expression has not been thoroughly exploited for identifying driver mutations and only a small fraction of published re-sequencing screenings of cancer genomes takes it into account to directly discriminate between driver and passenger mutations [43,60] or to assess the background mutation rate [61].

### Identification of novel drivers in ovarian carcinomas

**Figure 3:**
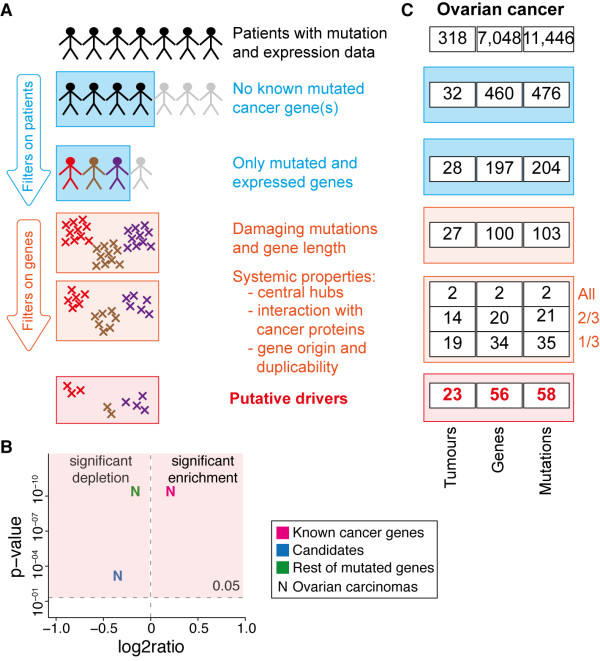
**Identification of novel driver genes in ovarian carcinoma**. (**A**) Pipeline to identify putative driver genes on the basis of patient and gene properties. Starting from all tumour samples with mutation and expression data, the first filters removes samples with mutations in known cancer genes and with mutated genes that are not expressed. Then, only short genes with damaging mutations are retained. Finally, genes with properties that resemble those of known cancer genes are identified as putative drivers. (**B**) Volcano plot for the expression of mutated genes in ovarian carcinomas. Of the 7,048 total mutated genes, only 4,723 had expression data. Of those, 223 were known cancer genes of the Cancer Gene Census [58]; 36 were previously defined as candidate cancer genes in ovarian cancer [11,62]; all remaining 4,464 mutated genes had no putative involvement in cancer. (**C**) Identification of novel drivers in ovarian carcinomas. Following our pipeline, we identified 56 genes that may favour cancer development in 23 ovarian cancer patients.

To identify novel cancer genes from mutation data, we developed an integrated pipeline that identifies putative drivers on the basis of the similarity between their properties and those of known cancer genes (Figure 3A). The starting point were cancer samples that underwent both sequencing and expression profiling, since we found that driver mutations occur in genes that are also expressed in the cancer tissue. As a first filter, we removed tumours with at least one known mutated and expressed cancer gene, because these genes are the most likely, albeit not the only, cancer drivers in these tumours. Since our main purpose was to prioritize the selection, we reasoned that it was more likely to find novel drivers in tumours with no mutations in known cancer genes. Further filters were then applied at the gene level. First, mutations were analysed for their putative effects on the encoded proteins, in order to eliminate passenger mutations with no functional consequences. Second, since a positive correlation between gene length and gene mutation frequency exists (Figure 1C), all genes in the top 5% of gene length (>4,450 bp) and mutated in more than five different cancer types (Figure 1A) were removed. Finally, four systems-level properties were evaluated to prioritize genes that resemble known cancer genes. We considered in particular high connectivity and centrality of the protein products in the human protein-protein interaction network [25,26]; direct interaction with a known cancer protein [20]; gene evolutionary appearance and duplicability. In the latter case, we prioritized genes that originated either early in evolution or with metazoans and vertebrates [29].

We applied our pipeline to 318 ovarian carcinomas with available sequencing and expression data that could be obtained from the Cancer Genome Atlas and used with no restrictions. Furthermore, all ovarian carcinomas underwent whole exome sequencing and matched expression profiling [62], therefore they constituted the ideal cases for our analysis. Before applying the pipeline for the selection of new drivers, we confirmed that also for this set of patients, similarly to other cancer types (Figure 2C), known cancer genes tend to be expressed in the tumour where they are mutated, while the rest of mutated genes are poorly expressed (Figure 3B and Additional file 1, Figure S3). The vast majority of the analysed ovarian carcinomas (286/318, 90% of the total) had at least one known cancer gene (mostly *TP53*) that was mutated and expressed and were therefore discarded from further analysis. After applying all other filters, we identified 58 putative driver mutations in 56 genes that were mutated and expressed in 23 of the 32 ovarian carcinomas with previously unknown genetics determinants (72%, Figure 3C).

To test the performance of our method in detecting known cancer drivers, we applied it to 130 of the 318 ovarian carcinomas that had mutations in 31 known tumour suppressor genes (Additional file 1, Figure S4). We correctly identified the mutated tumour suppressor genes as the cancer drivers in almost all tumours (123 out of 130 Additional file 2, Table S6). Furthermore, in the same samples we also identified additional putative drivers that are known to co-operate in tumour development. For example, in tumours where we found *TP53 *mutations, we also identified genes such as *CDH1 *and *CDKN2C *that often co-mutate with *TP53 *and are known to have synergic tumour-suppressor activity [63-66]. Therefore, in addition to pinpoint novel drivers, our method could also be applied to search for second hits or co-operating genes that help tumour development. In this respect one interesting putative co-driver is *NUMB*, a gene that encodes a negative regulator of NOTCH [67] and prevents TP53 ubiquitination and degradation [68]. The functional impairment of this gene upon damaging mutation might thus enhance tumour development because of the activation of the *NOTCH *oncogene and the degradation of *TP53 *tumour suppressor.

### Novel drivers of ovarian cancer resemble tumour suppressors and affect gene transcription, cell proliferation and survival

We had several indications that the mutated genes that we identified as putative drivers might indeed play an active role in ovarian carcinogenesis.

First, in addition to being all predicted as damaging by at least two out of three predictors (see Methods), 60% of the 58 mutations either modified protein functional domains or removed >50% of the protein sequence (Additional file 2, Table S7). Furthermore, the vast majority (77%) of the genomic sites where the mutation occurred are highly conserved among vertebrates (MultiZ score >0.95) [69] (Additional file 2, Table S7). Both these observations suggest a likely functional role of the mutations.

**Figure 4:**
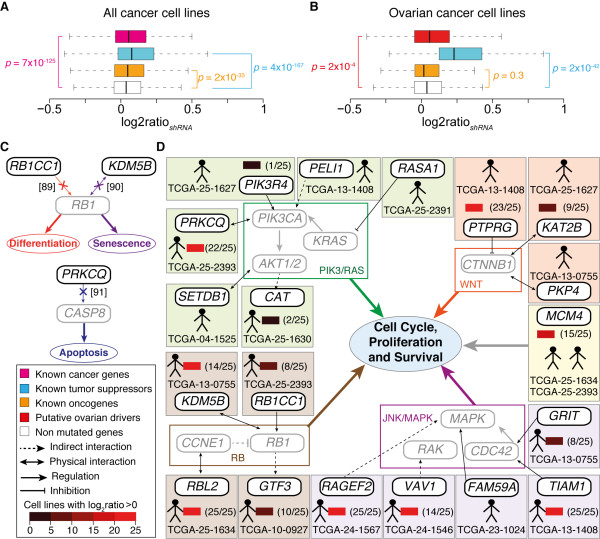
**Properties of putative drivers in ovarian cancer**. (**A**) Gene silencing effects of 395 known cancer genes with available shRNA data in 102 cancer cell lines. The distributions of log2ratios of the shRNA concentrations in the final cell population and the initial DNA pool (log2ratio*_shRNA_*, see Methods) were compared between known cancer genes, oncogenes, tumour suppressors and the non-mutated genes using Wilcoxon test. Complete data are reported in Additional file 2, Table S8. (**B**) Gene silencing effects of the 40 putative drivers identified with our pipeline, seven tumour suppressors and eight oncogenes with available shRNA data in 25 ovarian cancer cell lines. The list of known tumour suppressors and oncogenes associated with ovarian cancer was derived from the Cancer Gene Census [58]. Complete data are reported in Additional file 2, Table S9. (**C**) Confirming evidence of the effect of RNAi on three putative drivers. The block of *RB1CC1 *and *KDM5B *via RNAi leads to RB1 repression, with a consequent loss of the ability of RB1 to promote cell differentiation [92] and senescence [93], respectively. Interestingly, the Rb pathway is a known key player in ovarian cancer [62]. Similarly, anti-*PRKCQ *siRNAs inactivate CASP8. As a consequence, the CASP8/BCL10/MALT1 complex cannot be formed, thus preventing the cells to enter apoptosis [94]. (**D**) Effect of putative drivers on cell proliferation and survival. Reported are the links with pathways involved in gene proliferation of 19 out of 56 putative drivers mutated in 13 out of 23 tumour samples. The sample ID where the gene is mutated is provided together with the number of ovarian cancer cell lines over the total that displayed increased proliferation upon gene silencing, when available.

Second, we measured the effect of silencing the putative driver genes via RNA interference (RNAi), which mimics the effect of loss-of-function mutations and can therefore be used to infer the effect of gene impairment in cancer [70]. We derived large-scale gene silencing data from short hairpin RNA (shRNA) screens of approximately 11,000 genes in 102 cancer cell lines [71]. To check whether our assumption of an overall increased cell proliferation upon impairment of genes harbouring driver mutations was correct, we compared the gene silencing effect of known cancer genes with that of the rest of non-mutated human genes in all cell lines (see Methods). As expected, we observed that the silencing of known cancer genes, and in particular of tumour suppressors, favoured cell growth significantly more than non-mutated genes (Figure 4A, Additional file 1, Figure S5 and Additional file 2, Table S8, Wilcoxon test). We then analysed the silencing effect of the putative driver genes identified with our pipeline in the 25 ovarian cancer cell lines used in the screen [71]. Out of the 56 predicted driver genes, 40 were screened via RNAi and 35 of them led to increased cell proliferation in at least one ovarian cancer cell line (Table 2). Furthermore, their silencing effect overall resembled that of known tumour suppressors on the same ovarian cell lines (Figure 4B and Additional file 2, Table S9, Wilcoxon test). Thus, as expected, our selection procedure mainly identified tumour suppressor genes, since we retained putative damaging mutations that disrupt the protein function (Additional file 2, Table S7). For at least three of these genes (*RBICCI*, *KDM5B*, *PRKCQ*) we also found direct literature support that confirmed the effect of their impairment (Figure 4C). Interestingly, all three genes are strong candidate drivers of ovarian cancer (see below).

**Table 2:** Putative novel drivers in ovarian cancer

Ovarian carcinoma sample	Gene	Ovarian cell lines with increased proliferation	Pathway/Biological process	Reference
TCGA-04-1525	*SETDB1*	NA	PI3K/RAS	[95]
	
	*SIPA1L1*	NA	Cytoskeleton organisation	[96]

TCGA-09-2051	*SPTLC1*	1	MYC	[97]

TCGA-10-0927	*GTF3*	10	RB	[98]
	
	*SQRDL*	1	Amino acid metabolism	[99]

TCGA-13-0717	*GRIT*	NA	JNK/MAPK	[100]

TCGA-13-0755	*ANXA2*	2	Vesicle transport	[101]
	
	*FBLN1*	1	Cell adhesion	[97]
	
	*FEN1*	4	DNA repair	[102]
	
	*GSS*	3	Glutathione metabolism	[103]
	
	*KDM5B*	14	RB	[104]
	
	*MBNL1*	3	Splicing regulation	[105]
	
	*PKP4*	NA	WNT	[106]
	
	*U2SURP*	NA	WNT	[97]
	
	*WDR61*	NA	Chromatin regulation	[107]

TCGA-13-1408	*F11R*	0	Cell adhesion	[108]
	
	*PELI1*	NA	PI3K/RAS	[109]
	
	*PTPRG*	23	WNT	[110]
	
	*TAF12*	18	Transcriptional activation	[111]
	
	*TIAM1*	25	JNK/MAPK	[112]

TCGA-13-1506	*BTBD2*	NA	NF-kB (transcription)	[113]

TCGA-13-1510	*RIOK3*	7	Unknown	NA

TCGA-23-1024	*FAM59A*	NA	JNK/MAPK	[114]
	
	*PBX2*	22	Transcriptional activation	[115]

TCGA-24-1431	*ANKZF1*	NA	Unknown	NA
	
	*ATP1B1*	0	Ion transport	[116]

TCGA-24-1544	*CCDC93*	10	Unknown	NA
	
	*HDAC6*	1	Chromatin regulation	[117]

TCGA-24-1546	*SUGP1*	7	Splicing regulation	[99]
	
	*VAV1*	14	JNK/MAPK	[118]

TCGA-24-1567	*HTATSF1*	5	Splicing regulation	[119]
	
	*INADL*	NA	Cell-cell interactions	[120]
	
	*KAL1*	0	Cell adhesion	[121]
	
	*RAGEF2*	25	JNK/MAPK	[122]
	
TCGA-25-1623	*PSD3*	NA	Transcriptional activation	[123]

TCGA-25-1627	*KAT2B*	9	WNT	[124]
	
	*PIK3R4*	1	PI3K/RAS	[125]

	*TTF2*	25	Transcriptional repression	[126]

TCGA-25-1630	*APEX1*	9	DNA repair	[127]
	
	*ATG3*	NA	Autophagy	[128]
	
	*CAT*	2	PI3K/RAS	[129]
	
	*DCAF6*	NA	Transcriptional activation	[130]

TCGA-25-1633	*SASH3*	0	Antigen receptor signalling	[131]

TCGA-25-1634	*GSN*	5	TP53	[132]
	
	*MCM4*	15	G1/S transition	[133]
	
	*RBL2*	25	RB	[134]

TCGA-25-2391	*RASA1*	5	PI3K/RAS	[135]

TCGA-25-2393	*ANO1*	0	Ion transport	[131]
	
	*FUT1*	1	Antigen synthesis	[136]
	
	*MCM4*	15	G1/S transition	[137]
	
	*PRKCQ*	22	PI3K/RAS	[138]
	
	*RB1CC1*	8	RB	[121]
	
	*ZC3H14*	NA	Translation	[139]

TCGA-36-1570	*PSD3*	NA	Transcriptional activation	[140]

TCGA-36-1574	*NRCAM*	NA	Cell adhesion	[141]
	
	*NSMAF*	NA	NF-kB (transcription)	[142]
	
	*PASK*	24	Translation	[143]

TCGA-36-1577	*PRKD1*	1	Transcriptional activation	[144]

Shown are 23 ovarian carcinomas hosting 58 putative driver mutations in 56 genes. Two genes (*MCM4 *and *PSD3*) were mutated in two tumours. For 40 genes with available RNAi data [71], the number of ovarian cell lines where an increased in cell growth was observed upon gene silencing is shown. Thirteen carcinomas (57% of the total) bear mutations in pathways previously associated with ovarian cancer, and six of them (26% of the total) had alterations in transcription-related pathways. A detailed description of the effects of each mutation is shown in Additional file 2, Table S7.

Finally, we investigated the association of the 56 putative driver genes with pathways known to be involved in ovarian cancer onset. We found that 13 of the 23 tumours (57% of the total) harboured mutations in 19 genes belonging to pathways that control cell proliferation and survival, including the RB and PI3K/RAS signalling pathways, which are altered in 67% and 45% of ovarian cancers, respectively [62] (Figure 4D). RNAi data were available for 14 out of these 19 genes and in all cases gene silencing led to increased proliferation in at least one ovarian cancer cell line and the block of eight genes (*KDM5B*, *TIAM1*, *RAGEF2*, *PRKCQ*, *VAV1*, *PTPRG, RBL2 *and *MCM4*) favoured cell growth in the majority of cell lines (Figure 4D). Although for the remaining 10 tumours no such a direct link with ovarian cancer could be drawn, six of them had alterations in gene transcription and in other two cases a general association with cancer could be made (Table 2). Therefore, overall >90% of tumours harboured genomic alterations in pathways associated with cancer.

## Discussion

The central tenet of our study was that cancer driver mutations occur in genes with peculiar properties and, therefore, such properties can be used to identify novel cancer genes. For example we showed that cancer genes with an established driver role are usually expressed in the tissue where they are mutated, thus suggesting that mutations in genes that are not expressed are neutral or passenger. In support to our results, the vast majority of cancer somatic mutations have been shown to occur in genomic regions associated with repressive chromatin marks [72]. This indicates that indeed most cancer mutations are neutral and occur in transcription-silent regions of the genome.

In addition to expression profiles, we analysed the evolutionary, genomics and network properties of genes mutated in 32 ovarian cancer carcinomas with previously unknown genetics determinants. These tumours constitute only a small fraction of ovarian carcinomas (approximately 10% of the initial set) since the large majority of affected individuals bear mutations in known cancer genes, in particular in *TP53*. Although cancer is usually the outcome of the alteration of several genes and multiple drivers are required for cancer progression [73], we reasoned that focusing on tumours with no mutation in known cancer genes could increase the chances to find novel drivers. Furthermore, this would also help identifying a possible cause of cancer onset and development also in tumours that harbour rare mutations. With our approach we were indeed able to find 56 putative cancer genes in >70% of previously uncharacterized tumours, thus significantly reducing the fraction of patients with unknown cancer determinants. In the vast majority of cases, at least one of the putative drivers exerts a function in pathways that are altered in ovarian cancer. This confirms that the high heterogeneity of the cancer mutational landscape is reduced when considering biological processes rather than single genes [19].

As a comparison with our method, we investigated whether the 56 putative cancer genes had also been detected in the original study on the same set of ovarian carcinomas [62], which also identified possible cancer genes using a variety of approaches, from gene mutation frequency to pathway and network analysis [62]. Our list of putative drivers showed very poor overlap with the genes identified in the original study, mainly because the latter were for the vast majority already known cancer genes or had no expression data, and were therefore discarded from our analysis. Interestingly, some overlap existed between our list of 56 drivers and the network modules that were significantly mutated in ovarian cancer [24]. In particular, we identified five genes in common between the two lists. The silencing via RNA interference of three of these five genes (*VAV1*, *TAF12 *and *GTF3*) resulted in increased proliferation in at least 10 ovarian cancer cell lines. This strongly suggests a role of tumour suppression of these genes, and this is worth further experimental investigation.

## Conclusions

Our analysis showed that the integration of several sources of information allows the identification of rare cancer genes. This may be of particular utility in tumours with no known driver mutations or where frequency-based methods cannot be applied. However, we also showed that an integrated analysis may be useful for the identification of mutated genes that may cooperate in promoting tumour development. The poor overlap with previous findings in the same set of tumour samples demonstrates that our approach is complementary to frequency-based methods. The integration of several methods based upon different theoretical assumptions may therefore result in a better and more complete characterization of the mutational landscape of cancer.

## Methods

### Gene sets used in the analysis

To derive a dataset of unique human genes (that is, genes with a unique locus in the genome), 33,398 protein sequences were retrieved from RefSeq v.51 [74] and aligned to the human reference genome (hg19) using BLAT [75]. In case of multiple isoforms aligning to the same locus, only the longest was retained [26]. Only genes located on autosomal chromosomes and chromosome × were considered for further analysis, for a total of 19,009 unique human genes. Gene length was calculated as the coding portion of the longest isoform for each locus.

The dataset of 10,681 genes with at least one somatic non-synonymous mutation in cancer was collected from 39 mutational screenings of cancer tissues [2,4-18,35-57] (Table 1, Additional file 2, Table S1). Genes were grouped into three classes: (1) known cancer genes included all genes whose mutations or amplifications are known to be involved in tumorigenesis (Cancer Gene Census, frozen on 15 November 2011, and Census of Amplified Genes in Cancer) [58,59]; (2) candidate cancer genes that were found recurrently mutated in different tumour samples and, therefore, likely to harbour driver mutations (candidates were extracted directly from the corresponding experiments, Additional file 2, Table S1); (3) genes with low frequency non-synonymous mutations. The rest of human genes used for comparison were defined as all human genes with either no mutations or only synonymous mutations (Table 1).

### Expression of mutated genes in normal and cancer tissues

Expression data for 12,397 genes in 109 healthy tissues were derived from two microarray experiments on 36 [76] and 73 [77] normal human tissues, respectively, for a total of 109 unique tissues. The raw CEL files were downloaded from the corresponding series (GSE2361 and GSE1133) stored in the Gene Expression Omnibus (GEO) [78], normalised and analysed using the MAS5 algorithm included in the R affy package [79,80] (Additional file 2, Table S10). Given that more than one probe could be associated with a single gene, a gene was labelled as 'expressed' if at least half of the corresponding probes had detection *P *values <0.05. Housekeeping genes were defined as genes expressed in at least 98% of the tissues (107/109), while tissue-specific genes were expressed in <25% of the tissues (27/109).

To test whether the fraction of housekeeping mutated genes (known, candidates and rest of genes with non-synonymous mutations) was different from the fraction of housekeeping genes among the rest of human genes, Fisher's exact test with one degree of freedom was used. Fisher's test was used because of the small number of genes that were compared (only 10 candidate genes were housekeeping). The same test was applied to assess the differences in the fraction of tissue-specific genes between mutated and non-mutated genes.

To check whether mutated genes tend to be expressed in the corresponding healthy tissue, one or more of the 109 normal tissues with expression data were associated with the 20 tumour types with mutation data (Additional file 2, Table S3). For each of the three groups of mutated genes (known, candidates and rest of genes with non-synonymous mutations), the fraction of expressed genes over the total (*f_exp_mutated_*) was calculated in the tissues corresponding to each of the 20 tumour types. Similarly, the fraction of expressed non-mutated human genes in the same tissue (*f_exp_rest_*) was also measured and the two proportions were compared using chi-squared test with one degree of freedom to determine whether they were statistically different. Results were visualised as volcano plots that reported the log2ratios between the two fractions of expressed genes and the corresponding *P *value as measured with chi-squared test:

$$\text{log}2ratio={\text{log}}_{2}\frac{f_{\text{exp}\text{\_}mutated}}{f_{\text{exp}\text{\_}rest}}$$

To verify whether mutated genes were expressed at higher or lower levels than the rest of human genes, the median expression level was calculated in each of the 109 tissues. All genes with expression higher than the median were considered as highly expressed, while all genes with expression lower than the median were defined as lowly expressed. In each tissue, the fraction of highly expressed genes over the total in each of the three groups of mutated genes (*h_exp_mutated_*) and the fraction of highly expressed non-mutated genes (*h_exp_rest_*) were compared using the chi-squared test with one degree of freedom. Results were displayed as volcano plots that reported the log2ratio between the fractions of highly expressed mutated and non-mutated genes and the corresponding *P *value assessed with chi-squared test:

$$\text{log}2ratio={\text{log}}_{2}\frac{h_{\text{exp}\text{\_}mutated}}{h_{\text{exp}\text{\_}rest}}$$

For three of the 39 mutational screenings [36,43,44], both expression and mutation data were available for each analysed tumour sample. The raw CEL files were downloaded from GEO and the data were processed as described for the normal tissues (Additional file 2, Table S10). Since the study by Barretina *et al. *[36] reported the mutational screen of 722 genes and only a small number of mutations were detected in each sample, tumours were clustered into four groups, on the basis of the tumour subtype (Additional file 2, Table S4). A pipeline similar to that described for the analysis of normal tissues was applied to determine whether higher fraction of cancer genes were expressed in the cancer tissues where they were also mutated. Briefly, the fractions of expressed mutated and non-mutated genes in each tumour sample were compared using chi-squared test with one degree of freedom, in each sample. As for the other analyses, the results were displayed as volcano plots where each log2ratios of the fractions of expressed genes between mutated genes and non-mutated genes were displayed in association with the corresponding *P *values of the chi-squared test.

### Analysis of ovarian carcinoma samples

Genes mutated in ovarian carcinomas were derived from the Cancer Genome Atlas [81]. In addition to all validated somatic mutations (data level 3), the raw CEL files of the expression data corresponding to the same tumour sample were also retrieved (platform HG_U133A, data level 1, Additional file 2, Table S10). Of the 323 tumours, five were removed because they did not undergo whole exome sequencing. The fraction of expressed and mutated genes was calculated for each carcinoma as described above, and compared with the corresponding fraction of expressed and non-mutated human genes using the chi-squared test (one degree of freedom). Starting from the list of all mutated genes, several filters were applied to identify putative driver mutations (Figure 3A). First, carcinomas with mutations in at least one known cancer gene from the Cancer Gene Census [58] and those with no expression data for any mutated gene were discarded. Second, three different predictors (SIFT [30], Polyphen [31] and MutationTaster [32]) were applied to infer the effect of mutations. Only frameshift, nonsense and splice-site mutations, as well as missense mutations predicted as damaging by two out the three predictors (SIFT score >0.95, Polyphen score >0.9, or labelled as 'disease causing' by MutationTaster [82]) were retained. Third, the gene length of the coding portion was taken into account and all genes in the bottom 95% of gene length were retained (coding length <4,450 bp). Genes longer than 4,450 bp were retained only if mutated in less than five different cancer types. This filter discarded genes that mutate at high frequency because of their length. Finally four systemic properties were investigated: protein connectivity and centrality in the protein-protein interaction network; interaction(s) with known cancer proteins; evolutionary origin; and duplicability. To measure protein connectivity and to determine the occurrence of direct interactions with known cancer proteins, data on 98,492 experimentally proven protein-protein interactions between 13,531 human proteins were integrated from five databases (HPRD [83], BioGRID [84], IntAct [85], MINT [86] and DIP [87]), as previously described [88]. The *IGRAPH *module for R [89,90] was used to measure degree, betweenness and direct interactions with known cancer proteins. Central hubs were defined as the 25% most connected (degree >14) and most central (betweenness >9,198) proteins of the network. Evolutionary origin and gene duplicability were defined as previously described [29]. Briefly, gene origin was traced as the most ancient node of the tree of life where orthologs for a given human gene could be found. A gene was defined as duplicated if at least one human paralog was present in the corresponding cluster of orthologs, otherwise it was considered as singleton. All scripts used to run this pipeline are available as Additional file 3.

### Effect of gene silencing on cell proliferation using RNA interference

Short hairpin RNA (shRNA) data were derived from the high throughput analysis on 10,941 genes (corresponding to 52,209 probes) in 102 cancer cell lines (including 25 ovarian cancer cell lines) and analysed as described in the original study [71], with slight modifications. Briefly, the raw GCT file with the measurements of the shRNA abundance in all cell lines (20110303_achilles2.gct) was downloaded and normalized to obtain the corresponding shRNA score for each gene probe. The effect of the individual gene silencing on cell proliferation was calculated in comparison with the initial DNA pool, using an in-house modified version of the R *shRNAscores *package from the Integrative Genomics Portal at the BROAD Institute [91]. In order to determine the silencing effect of each gene, the concentration of its corresponding shRNA in the final cell population and the initial DNA pool was compared. To have a single comparison for each gene probe *i*, the log2ratio was calculated between the means of all replicates in each cell line and the means of replicates in the initial DNA pool:

$$\text{log}2ratio_{shRNA.h.i}={\text{log}}_{2}\left[\frac{\frac{1}{m}\overset{m}{\underset{j=1}{\sum }}shRNA\text{\_}score_{h,i,j}}{\frac{1}{n}\overset{n}{\underset{k=1}{\sum }}shRNA\text{\_}score_{DNA,i,k}}\right]$$

Where *m *and *n *are the number of replicates in the considered cell line *h *and in the reference DNA pool, respectively. Having a median of five probes associated with a single gene, only the top-scoring shRNA value among all probes was considered as the representative effect of that gene on cell proliferation in order to minimise the false positives [71]. The ratio was preferred to the difference between cell lines and DNA pool (as in the original paper [71]) in order to better appreciate the modifications in the cell proliferation caused by gene silencing. To measure the overall effect on gene proliferation of the silencing of known cancer genes, the log2ratio*_shRNA _*distributions between 395 genes (95 tumour suppressors and 300 oncogenes) from the Cancer Gene Census with at least one shRNA probe and the rest of 10,546 non-mutated genes in all 102 cancer cell lines were compared (Figure 4A). Shapiro-Wilk test was applied to control for the shape of the distributions. Since the distribution could not be considered as normal (*P *value <10^-50^, Additional file 1, Figure S5), Wilcoxon test was used to assess the differences between them. For the analysis on ovarian cancer, only the 25 ovarian cancer cell lines and 15 known cancer genes (seven tumour suppressors and eight oncogenes) that were associated with ovarian cancer in the original annotation of the Cancer Gene Census were considered (Figure 4B).

## Authors' contributions

MDA performed all analyses; FDC conceived the study; MDA and FDC wrote the manuscript. All authors read and approved the final manuscript.

## Supplementary Material

Additional file 1 **Supplemental figures**. This file contains Figures S1-S5. Click here for file

Additional file 2 **Supplemental tables**. This file contains Tables S1-S10. Click here for file

Additional file 3 **Scripts to identify putative drivers**. This file contains a collection of scripts to run the pipeline for the identification of cancer drivers. Click here for file
