# Supplementary material for: Integrated analysis of recurrent properties of cancer genes to identify novel drivers
Source: Genome Biol. 2013 May 29;14(5):R52. doi: 10.1186/gb-2013-14-5-r52 (PMC4054099; doi:10.1186/gb-2013-14-5-r52)
Supplement: Additional file 1 — Supplemental figures. This file contains Figures S1-S5. [file gb-2013-14-5-r52-S1.PDF]

Additional file 1

Supplemental figures

Figure S1 - Distribution of expression breadth for all human genes

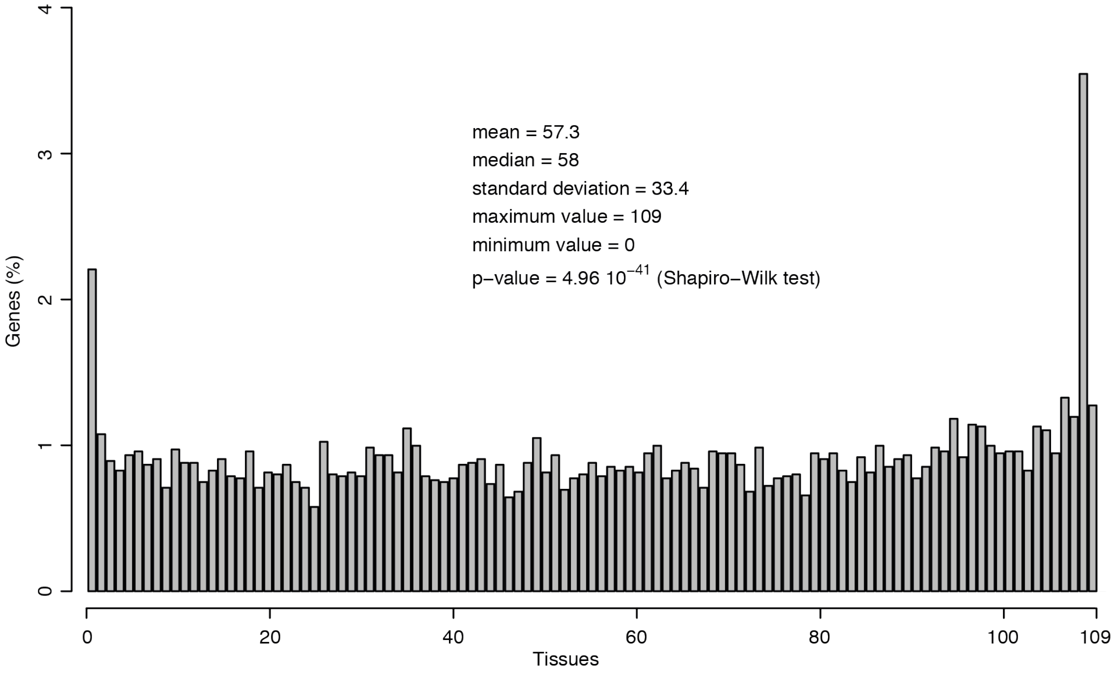

Shown are the fractions of genes expressed in one to 109 human tissues, as reported in [1, 2]. P-value from Shapiro-Wilk test assessed that the distribution is not normal, therefore Wilcoxon test was used to measure the differences between cancer genes and the rest of human genes (Figure 2A).

Figure S2 - Expression of known, candidate and rest of mutated genes in normal tissues

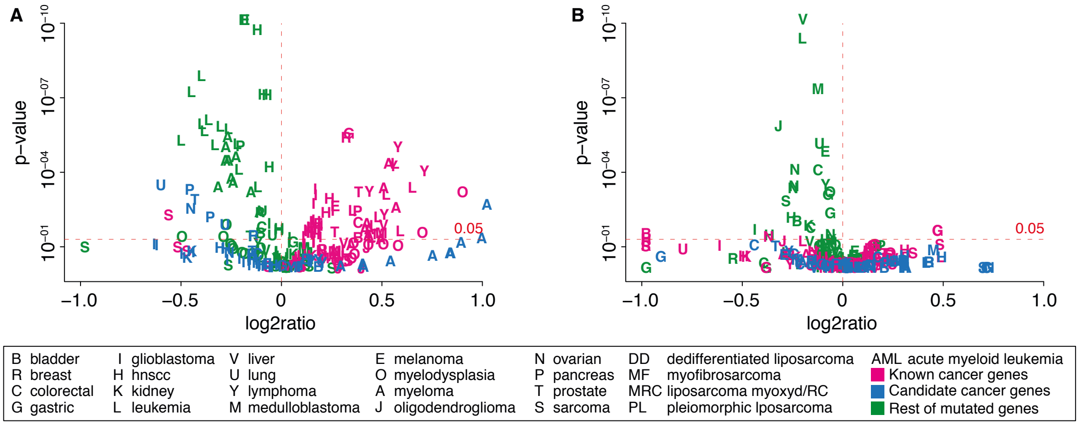

(A) Volcano plot for the expression of mutated genes in each single tissue (Table S2). For each cancer type one or more points are represented, depending on the number of tissues associated with it. Log2ratios were calculated between the fraction of expressed mutated genes in each tissue and the corresponding fraction of the rest of human genes. (B) Volcano plot for the expression levels of mutated genes in each single tissue. Log2ratios were calculated between highly expressed mutated genes and highly expressed non-mutated genes. P-values refer to chi-squared test. The volcano plots corresponding to the overall expression in all tissues for each cancer type are shown in Figure 2.

Figure S3 - Expression of known, candidate and rest of mutated genes in ovarian carcinomas

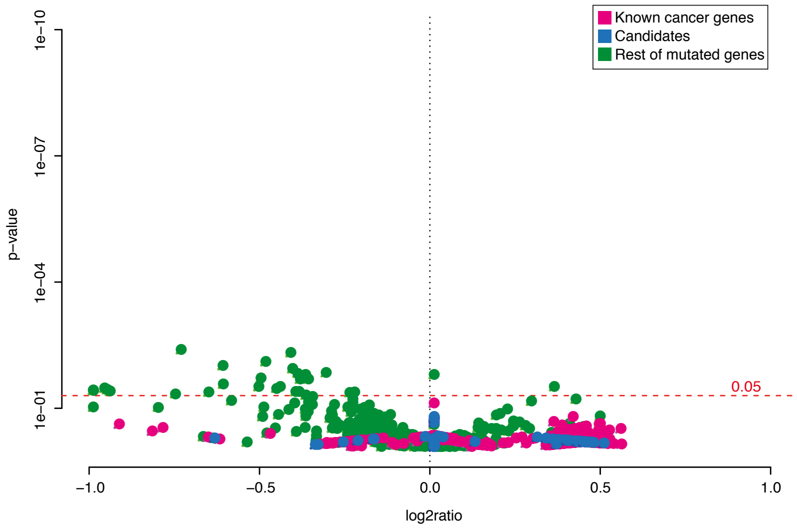

Volcano plot for the expression of mutated genes in each of the 318 ovarian cancer patients. Of the total 12,378 genes with expression data, 223 were known cancer genes of the Cancer Gene Census [3], 36 were ovarian-specific candidate cancer genes [4, 5] and 4,464 had non-synonymous mutations but no putative role in tumorigenesis. Log2ratios were calculated between expressed mutated genes and expressed non-mutated genes. P-values refer to chi-squared test. The volcano plots corresponding to the overall expression of all genes for each cancer type are shown in Figure 3B.

Figure S4 - Identification of driver mutations in known tumour suppressor genes

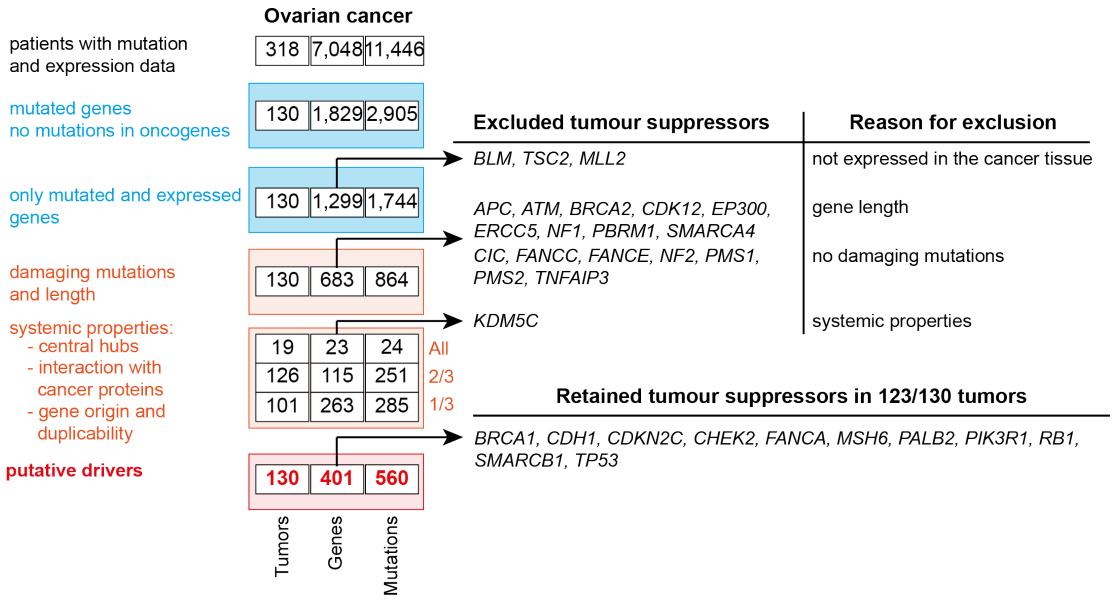

130 tumours with at least one mutated tumour suppressor gene but no mutated oncogenes were selected from the 318 carcinomas with both mutation and expression data, for a total of 1,829 mutated genes, of which 31 tumour suppressors. In 123 of all tumours (95% of the total) the tumour suppressor was identified as the driver (Table S6). Tumour suppressor genes that were excluded at each step of the pipeline as well as those that were retained are also shown. Since also other putative drivers were identified in each tumor, they may act as co-operating genes.

Figure S5 – Distribution of log2ratio$_{shRNA}$ values of all human genes in 102 cancer cell lines

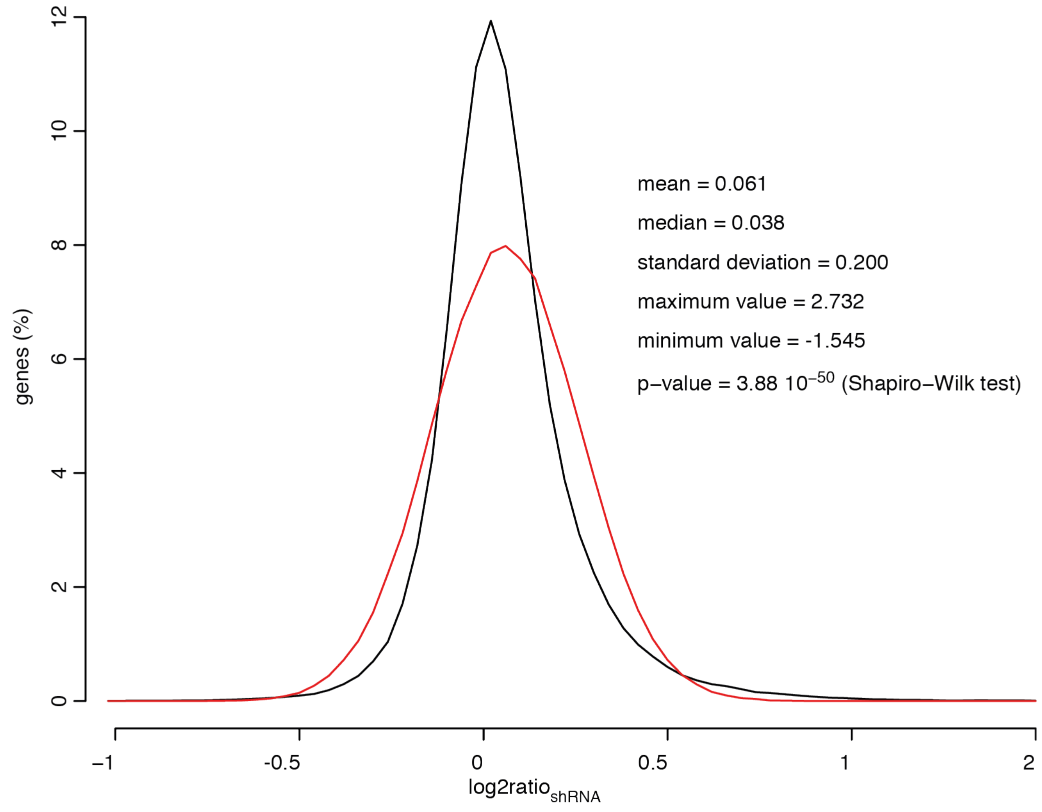

The fraction of 10,941 human genes with a given log2ratio$_{shRNA}$ in 102 cancer cell lines [6] is shown (black). The red line displays the corresponding normal distribution with the same mean and standard deviation. Shapiro-Wilk test demonstrates that the distribution is not normal, therefore Wilcoxon test was used to compare cancer genes with the rest of human genes (Figure 4A,B).

References

1. Ge X, Yamamoto S, Tsutsumi S, Midorikawa Y, Ihara S, Wang SM, Aburatani H: **Interpreting expression profiles of cancers by genome-wide survey of breadth of expression in normal tissues**. *Genomics* 2005, **86**(2):127-141.
2. Su AI, Wiltshire T, Batalov S, Lapp H, Ching KA, Block D, Zhang J, Soden R, Hayakawa M, Kreiman G *et al*: **A gene atlas of the mouse and human protein-encoding transcriptomes**. *Proc Natl Acad Sci U S A* 2004, **101**(16):6062-6067.
3. Futreal PA, Coin L, Marshall M, Down T, Hubbard T, Wooster R, Rahman N, Stratton MR: **A census of human cancer genes**. *Nat Rev Cancer* 2004, **4**(3):177-183.
4. Bell D, Berchuck A, Birrer M, Imielinski M, Chien J, Cramer DW, Dao F, Levine DA, Olvera N, Dhir R *et al*: **Integrated genomic analyses of ovarian carcinoma**. *Nature* 2011, **474**(7353):609-615.
5. Kan Z, Jaiswal BS, Stinson J, Janakiraman V, Bhatt D, Stern HM, Yue P, Haverty PM, Bourgon R, Zheng J *et al*: **Diverse somatic mutation patterns and pathway alterations in human cancers**. *Nature* 2010, **466**(7308):869-873.
6. Cheung HW, Cowley GS, Weir BA, Boehm JS, Rusin S, Scott JA, East A, Ali LD, Lizotte PH, Wong TC *et al*: **Systematic investigation of genetic vulnerabilities across cancer cell lines reveals lineage-specific dependencies in ovarian cancer**. *Proc Natl Acad Sci U S A* 2011, **108**(30):12372-12377.
